# Genome wide association study and genomic risk prediction of age related macular degeneration in Israel

**DOI:** 10.1038/s41598-024-63065-0

**Published:** 2024-06-06

**Authors:** Michelle Grunin, Daria Triffon, Gala Beykin, Elior Rahmani, Regev Schweiger, Liran Tiosano, Samer Khateb, Shira Hagbi-Levi, Batya Rinsky, Refael Munitz, Thomas W. Winkler, Iris M. Heid, Eran Halperin, Shai Carmi, Itay Chowers

**Affiliations:** 1 Braun School of Public Health and Community Medicine, The Hebrew University of Jerusalem, POB 12271, 9112102 Jerusalem, Israel; 2 Department of Ophthalmology, Hadassah-Hebrew University Medical Center, POB 12000, 91120 Jerusalem, Israel; 3 Department of Computational Medicine, University of California, Los Angeles, Los Angeles, CA USA; 4 Molecular Microbiology and Biotechnology, Tel Aviv University, Tel Aviv, Israel; 5 Department of Anesthesiology, University of California, Los Angeles, Los Angeles, CA USA; 6 Department of Human Genetics, University of California, Los Angeles, Los Angeles, CA USA; 7 Department of Genetic Epidemiology, University of Regensburg, Regensburg, Germany; 8 Department of Genetics, University of Cambridge, CB21TN Cambridge, UK

**Keywords:** Macular degeneration, Genetics, Genetic association study, Population genetics

## Abstract

The risk of developing age-related macular degeneration (AMD) is influenced by genetic background. In 2016, the International AMD Genomics Consortium (IAMDGC) identified 52 risk variants in 34 loci, and a polygenic risk score (PRS) from these variants was associated with AMD. The Israeli population has a unique genetic composition: Ashkenazi Jewish (AJ), Jewish non-Ashkenazi, and Arab sub-populations. We aimed to perform a genome-wide association study (GWAS) for AMD in Israel, and to evaluate PRSs for AMD. Our discovery set recruited 403 AMD patients and 256 controls at Hadassah Medical Center. We genotyped individuals via custom exome chip. We imputed non-typed variants using cosmopolitan and AJ reference panels. We recruited additional 155 cases and 69 controls for validation. To evaluate predictive power of PRSs for AMD, we used IAMDGC summary-statistics excluding our study and developed PRSs via clumping/thresholding or LDpred2. In our discovery set, 31/34 loci reported by IAMDGC were AMD-associated (P < 0.05). Of those, all effects were directionally consistent with IAMDGC and 11 loci had a P-value under Bonferroni-corrected threshold (0.05/34 = 0.0015). At a 5 × 10^−5^ threshold, we discovered four suggestive associations in *FAM189A1*, *IGDCC4*, *C7orf50*, and *CNTNAP4*. Only the *FAM189A1* variant was AMD-associated in the replication cohort after Bonferroni-correction. A prediction model including LDpred2-based PRS + covariates had an AUC of 0.82 (95% CI 0.79–0.85) and performed better than covariates-only model (P = 5.1 × 10^−9^). Therefore, previously reported AMD-associated loci were nominally associated with AMD in Israel. A PRS developed based on a large international study is predictive in Israeli populations.

## Introduction

Age-related macular degeneration (AMD) is the leading cause of blindness in the elderly population. The risk for developing AMD is strongly associated with the genetic background of the individual^1,2^. In 2005, AMD was the first disease for which genome-wide association studies (GWASs) have identified risk variants^3,4^. Via a seminal paper published in 2016, the International Age-Related Macular Degeneration Genomics Consortium (IAMDGC) has reported the genotyping of more than 30,000 AMD patients and controls of European ancestry and the discovery of 52 risk variants across 34 loci^2^.

Israel is home to a number of populations of distinct genetic ancestry, including Ashkenazi Jews, non-Ashkenazi Jews—predominantly North-African Jews and Middle-Eastern Jews, and Arabs—predominantly Palestinians, Bedouins, and Druze. These populations are genetically diverse, having genetic ancestry related to the Middle East, Africa, and Europe, with variable admixture proportions^5–8^. Some of the populations have experienced recent population-specific genetic drift due to founder events and endogamy^7,9,10^. The unique genetic background of the Israeli populations suggests that the genetic architecture of AMD might be different in these populations compared to Europeans. In addition, the Israeli populations that have experienced strong genetic drift may harbor deleterious risk variants at a considerable frequency. This will increase power for discovering novel risk variants^11^ as previously observed for other retinal diseases^12–14^.

Previous studies of the genetic basis of AMD in Israel found that the most prominent risk variants—the genes *CFH*^15^ and *HTRA1/ARMS2*^16^—were associated with AMD. However, the *C2* locus, one of the top risk loci worldwide, was not associated with AMD in Israel^17^. The 2016 study of the IAMDGC included an Israeli cohort. However, it was analyzed jointly with the other studies, which was uninformative about Israeli-specific genetic architecture and risk variants. Searching for population-specific risk variants is important even beyond the population under study, as any discovered variants and biological pathways may provide insight into the pathogenesis of the disease.

Polygenic risk scores (PRSs) were recently developed for numerous diseases based on the results of large-scale GWASs^18^. Conceptually, a PRS is the count of risk alleles carried by an individual, where each allele is weighted by its effect size (usually the log odds-ratio), as estimated by GWAS. In practice, the list of single-nucleotide polymorphisms (SNPs) that are included in the score is optimized based on their strength of association (e.g., comparing multiple P-value cutoffs) and their correlation with other SNPs, in some methods accounting for prior assumptions on the genetic architecture of the disease^19^. While PRSs cannot unambiguously distinguish healthy and affected individuals (due to the small proportion of variance in disease liability they explain), individuals at the top PRS quantiles are at a particularly high risk^20,21^. These individuals can then be subjected to personalized screening or prevention.

A number of recent papers have developed or examined PRSs for AMD, showing that the PRS has considerable power to predict disease status and disease progression^2,22–27^. However, it is known that PRS accuracy can substantially decrease when evaluated in populations or ancestries other than the ones used for the original GWAS (usually European populations and ancestries)^28,29^. So far, no study has examined the accuracy of an AMD PRS in any of the Israeli sub-populations, which forms a barrier to the implementation of DNA-based risk stratification.

In this paper, we used data on 558 AMD cases and 325 controls to investigate the genetic basis of AMD in the Israeli populations. Our study had three main goals. (1) To determine whether previously identified risk variants (from the IAMDGC 2016 GWAS) are associated with AMD in Israel, either across all Israeli sub-populations or in a population-specific manner. (2) To discover putative new AMD risk variants by running a GWAS in the Israeli study, anticipating that despite the small sample size, we may be able to identify risk variants that have drifted to high frequencies in the Israeli founder populations. (3) To evaluate the accuracy in the Israeli population of a PRS generated based on the IAMDGC GWAS. We show that the vast majority of previously discovered risk variants are also associated with AMD in Israel, Accordingly, a PRS based on previously discovered variants has high predictive power. While our study was too small for discovering new risk variants at a genome-wide significance level, our study suggested a number of putative associations at an attenuated significance threshold.

## Results

### Replication of known AMD loci

A previous large-scale AMD GWAS by the IAMDGC (n = 33,976^2^) has discovered 34 associated loci. We examined the association of these loci with AMD status in our Israeli discovery set (403 AMD cases and 256 controls). Using the SNP with the lowest P-value in each locus, we found that most loci (31/34) were associated with AMD at a nominal significance level of P < 0.05 with a direction of effect consistent with that of the IAMDGC (Supplementary Tables 2 and 3). The number of loci associated at the Bonferroni correction threshold (0.05/34 = 0.0015) was 11/34 (Supplementary Table 2). The top ranked loci were* CFH* (P = 1.6 × 10^−9^) and nearby loci on chr1, and *ARMS2*/*HTRA1* (P = 3.4 × 10^−9^, 5.1 × 10^−9^, respectively). The next significant locus was near *SYN3* (P = 5.7 × 10^−5^). Association statistics for the known AMD risk loci for Ashkenazi Jews (AJ) (242 cases and 136 controls) and Arabs (36 cases and 30 controls) are reported in Supplementary Tables 4 and 5. We note that replication was to some extent expected, given that the majority of the Israeli cohort was included in the IAMDGC. However, the same 34 loci were associated with AMD at genome-wide significance even when all Israeli samples were excluded from the IAMDGC GWAS.

### Discovery GWAS

We next ran a GWAS in our discovery set (AMD cases: n = 403, controls: n = 256). No novel variant was associated at the genome-wide significance threshold of 5 × 10^−8^. Setting a more liberal threshold of 5 × 10^−5^, and excluding variants in known risk loci, we identified four suggestive associations in the genes *C7orf50*, *IGDCC4*, *FAM189A1*, and *CNTNAP4* (Table 1; Fig. S2). None of these SNPs were associated with AMD in the IAMDGC data (P ≥ 0.04, Table 1). The variant rs116928937 in *IGDCC4* is exonic. Its allele frequency in European Americans was 1.23% (in the Exome Variant Server (NHLBI GO Exome Sequencing Project (ESP), Seattle, WA (URL: http://evs.gs.washington.edu/EVS/))), compared to 2.66% here. It is a missense variant (c.3188G>T), and according to Polyphen^30^ it is "probably-damaging”.

**Table 1:** SNP association with AMD in Israel Discovery Cohort.

SNP ID	Genomic position (hg19)	Gene	P-value (entire cohort)	Odds ratio (95% confidence interval) Entire cohort, AJ subset	Minor allele frequency (cases/controls)	Minor allele frequency (gnomAD NFE/AJ)	P-value in IAMDGC—leave one out	Replication P-value Genotype (Fisher's exact)
rs12701455	chr7:1055409	C7orf50	5.05 × 10–6	0.5 (0.26–94)	0.147/0.251	0.309/0.212	0.78	0.028
rs116928937	chr15:65677446	IGDCC4	3.73 × 10–6	0.13 (0.02–0.83), 0.07	0.008/0.055	0.0185/0.0064	0.89	1
rs1506825	chr16:76483019	CNTNAP4	6.91 × 10–6	0.57 (0.34–0.96)	0.4373/0.5703	0.455/0.469	0.04	0.85
rs1195500	chr15:29,687,047	FAM189A1	2.265 × 10–5	0.51 (0.27–0.96), 0.37	0.129/0.227	0.26/0.15	0.27	0.00001
rs11689931 (AJ specific)	chr2:206440979	PARD3B	3.19 × 10–6 for AJ	0.07	0.20/0.37 in AJ	0.301/0.306		Was not tested

Statistics for the association of SNPs with AMD status in our Israeli discovery cohort. Genomic coordinates are in hg19. The gene is the nearest to the SNP. Allele frequencies were computed in gnomAD, for either non-Finnish Europeans (NFE) or Ashkenazi Jews (AJ). The first four rows provide details on four SNPs associated with AMD in our discovery cohort with P-value < 5 × 10^−5^. The last row presents details on the top-associated SNP in the AJ subset of our cohort.

We attempted to replicate the association of these four loci in a replication set of n = 155 AMD cases and n = 69 controls (Supplementary Table 1). We applied a Bonferroni corrected threshold of 0.05/4 = 0.0125. Only the SNP in *FAM189A1* (rs1195500, chr15:29687047) replicated in the Israeli population (P < 0.0001 in Fisher’s exact test in both genotype and allele testing). The SNP rs12701455 (*C7orf50*) attained a P-value of 0.029 in the genotype-based test (Supplementary Table 1).

### Evaluating a polygenic risk score for AMD

We developed polygenic risk scores (PRSs) for AMD in Israel based on the results of the IAMDGC GWAS with the Israeli samples excluded (“leave-one-out”) and using two methods. The first method is clumping and thresholding (C + T), in which the most strongly associated SNP is retained from each LD block, as long as its P-value is under a threshold. The second method is LDpred2, which accounts for the influence of LD on effect sizes and incorporates a non-zero prior probability for having null effects. We generated nine C + T PRSs, corresponding to different P-value thresholds (exponentially increasing between 5 × 10^−8^ and 1), and four LDpred2 PRSs, corresponding to different values of the proportion of SNPs with non-zero effects and a sparsity parameter. For each PRS, we used logistic regression to predict AMD status based on age, sex, the first two principal components (a proxy of ancestry), and the PRS. We also fit a logistic regression model with covariates only. We used fivefold cross-validation to evaluate the accuracy of the various models, which we quantified using AUC (the area under the receiver operator characteristic curve (ROC curve)).

**Figure 1:**
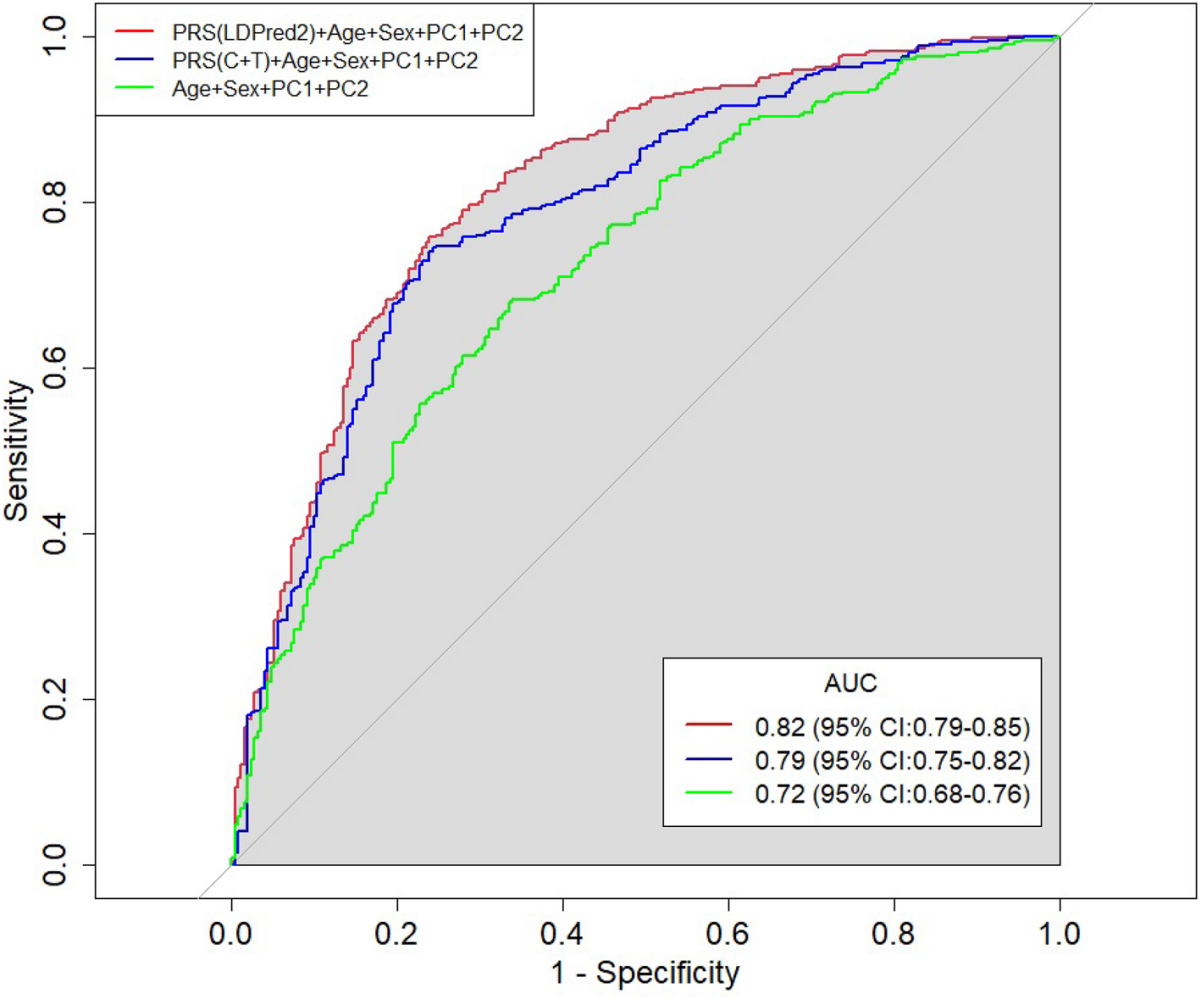
Prediction accuracy of selected PRS models. We show the ROC curve for the following AMD prediction models: the top C + T PRS (Fig. S3) + covariates (age, sex, PC1, and PC2); the top LDpred2 PRS + covariates; and a covariates-only model. The C + T PRS parameters were r^2^ > 0.5 and P < 5 × 10^−8^, and the LDpred2 PRS parameters were *P* = 0.056 and sparse = TRUE. The AUC estimates (after cross-validation) are indicated on top of the plot.

We compare the ROC curves of the most accurate (highest AUC) C + T model, the most accurate LDpred2 model, and the covariates-only model in Fig. 1. For C + T, the AUC was highest (0.79; 95% confidence interval (CI) 0.75–0.82) for the most stringent P-value threshold (5 × 10^−8^), for a PRS that included 360 variants. Interestingly, the AUC showed a trend (not statistically significant) of decreasing with increasing P-value thresholds (Fig. S3). The best LDpred2 model (parameters *P* = 0.056 and sparsity on) had a slightly higher AUC (0.82; 95% CI 0.79–0.85) than the best C + T model. The covariates-only model had a significantly lower AUC (0.72; 95% CI 0.69–0.76). This was also confirmed by DeLong's test for two correlated ROC curves (P = 5.1 × 10^−9^). Overall, our results suggest that including the PRS in the prediction model improves accuracy.

**Figure 2:**
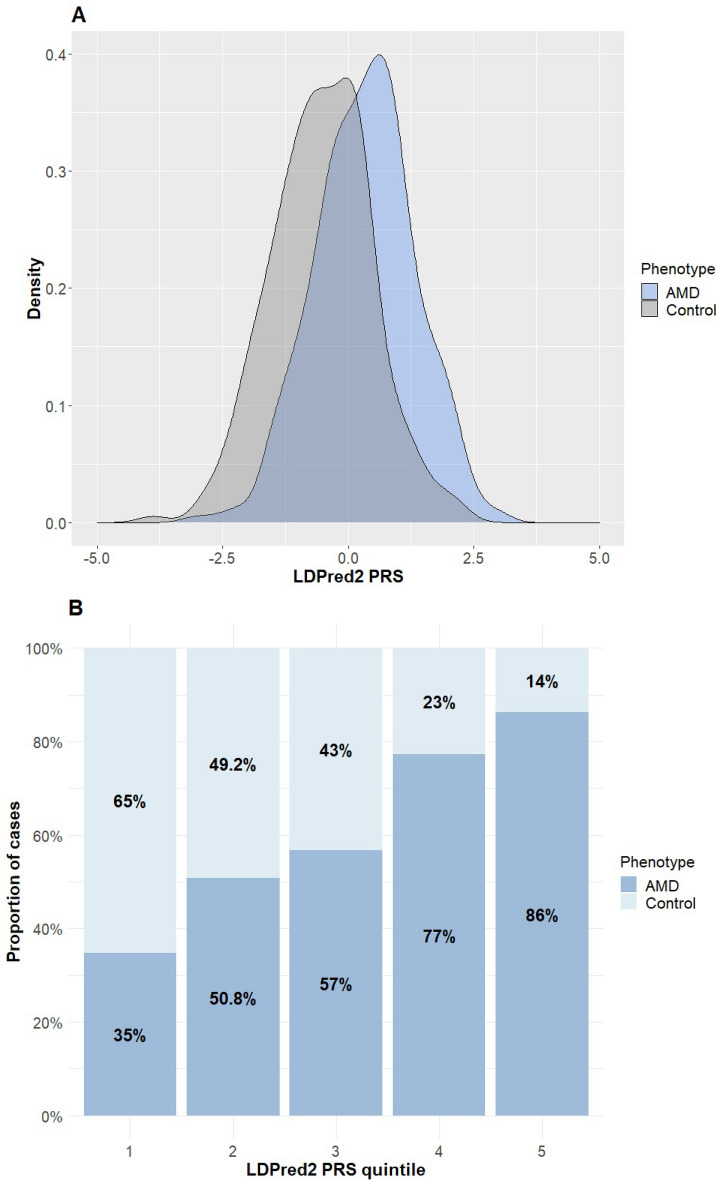
Comparing the PRS between cases and controls. (**A**) The density of the top LDpred2 PRS (after regressing out the first two principal components) in AMD cases and controls in our study (n = 403 and 259, respectively). (**B**) The proportion of AMD cases in our study by PRS quintiles. We again used the top LDpred2 PRS.

We show the distribution of the best LDpred2 PRS in cases and controls in Fig. 2A. The PRS distribution is different between cases and controls; however, considerable overlap exists. In Fig. 2B, we plot the proportion of cases in each quintile of the LDpred2 PRS, demonstrating that the proportion of cases steadily increases with increasing quintiles. Finally, we used Spearman's correlation test to assess the correlation between age at diagnosis (measured here as age at blood draw) and the PRS among AMD cases. In Fig. S4, we show a modest, yet significant, negative correlation between the variables (ρ = − 0.18, P-value = 0.0003, using the best LDpred2 PRS), suggesting that the PRS may be associated not only with disease status but also with age of onset.

## Discussion

In this work, we studied the genetic basis of AMD in the Israeli populations. We confirmed that most of the known risk loci for AMD, as previously identified in a large international study, are also associated with AMD in Israel. This suggests that the genetic architecture of AMD is similar between the Israeli and other populations. We then performed a genome-wide association study in our cohort in an attempt to identify novel risk variants. As expected due to the small size of our cohort, no novel variants were detected at the genome-wide significance threshold. Setting a more relaxed threshold of 5 × 10^−5^, we identified four suggestive variants.

One of the suggestive variants (rs1195500, in *FAM189A1*) replicated, after Bonferroni correction, in a small second set of cases and controls. *FAM189A1* is expressed in the brain (the Human Protein Atlas^31^ (https://www.proteinatlas.org/ENSG00000104059-FAM189A1/tissue) and GTEx^32^) and in normal human retina (the Retinal Transcriptome data^33^), but its function is yet unknown. The FAM189A1 gene is uncharacterized, and as such the function of its proteins is unknown. FAM189A1 was recently discovered to have rare pathogenic variants in neurodevelopmental disorders, and is present in neuronal cells of the brain^34^. Additional studies will be required to replicate the association of this variant as well as validate the involvement of *FAM189A1* in AMD.

*C7orf50* encodes for a newly discovered hormone, cholesin, that is secreted from the intestine in response to cholesterol absorption. This very recent discovery indicated that this gene, *C7orf50* is in charge of cholesterol synthesis in the liver, and is responsible for a reduction in circulating cholesterol levels^35^. This may be related to other cholesterol genes found in AMD^2^, as well as AMD’s connection to dietary cholesterol.

The two other suggestive genes, IDGCC4 and CNTNAP4, have been characterized. IGDCC4 belongs to the immunoglobulin superfamily. This protein might have a role in regulating the immune system against "para-inflammatory" diseases like AMD^36^, but no studies clearly showed its immune system role. It was present in a study discussing methylation patterns in lung adenocarcinoma in smokers, and was methylated in advanced tumors (stage II–IV) as compared to early, stage I tumors^37^. Methylation patterns in neonates in this gene were associated with higher birth weight, as well as with higher maternal BMI and glucose levels, indicating that it also plays a role in metabolism, both at birth and at later life^38^. CNTNAP4 is part of the contactin associated protein family (otherwise known as Caspr4), and is involved in both the development of myelinated axons and in stability of neuronal connections^39,40^. There is a known relationship between CNTNAP4 and neurodegenerative diseases, and a CNV variant in this gene was found to be inversely associated with healthy aging. The CNV variant was found to be protective against cognitive impairment, Alzheimer’s' and Parkinson’s, giving it a connection with normal neurological function^41^. Further investigation would be needed in order to ascertain their significance in the pathogenesis of AMD.

While our sample size is relatively small, it is, with 659 individuals in the base study and another 224 for replication, the largest AMD GWAS in Israel to date. While this was insufficient to discover any new associations at a genome-wide significance, our study was sufficiently powered to replicate most of the previously discovered associated loci. It was also sufficiently powered to detect an association between AMD status and a polygenic risk score, even when adjusting for standard covariates.

The evaluation of the AMD PRS in our Israeli cohort suggested multiple conclusions. First, the LDpred2 PRS had relatively high accuracy (AUC = 0.82), significantly better compared to not including the PRSs (Fig. 1). Second, with the simple clumping and thresholding approach, accuracy increased as more stringent P-value thresholds were used (Fig. S3). This could indicate that AMD is not as polygenic as other diseases. Third, as expected^42,43^, LDpred2 performed better than the C + T approach (Fig. 2A). Finally, high AMD PRS in our study associated not only with disease risk, but also with a lower age of onset (Fig. S4), as seen for other diseases such as colorectal cancer or cardiovascular disesase^44,45^. Prospective studies will be required to further validate this finding.

Our results for high predictive power of the AMD PRS are in line with previous studies^2,46^ in individuals of European ancestry. The transferability of the PRS to the Israeli population is perhaps expected given that the majority of our subjects had Ashkenazi Jewish ancestry, and given that PRSs for other diseases and traits were shown to have high accuracy in Ashkenazi Jews^47–50^. The transferability of PRSs into Ashkenazi Jews may be due to the high percentage of European ancestry in this population^7^. It is also consistent with our replication of the known risk loci. However, a limitation of our study is that it does not allow a direct head-to-head comparison of the strength of the PRS association with AMD between our Israeli cohort and cohorts of individuals of European ancestry. Our sample size was also too small to evaluate the PRS accuracy in other sub-populations, which could be the goal of future studies. Further improvement of the PRS may be achieved via denser genotyping or larger and more diverse imputation reference panels. Additionally, multiple methods can leverage even small samples from a target non-European population to improve a PRS constructed using large GWASs in Europeans^51,52^. However, such efforts will require additional samples for evaluation of the resulting PRSs.

## Materials and methods

Our discovery set consisted of 403 AMD cases and 256 controls (659 total) recruited at Hadassah Medical Center, as previously reported^53^. Our cases included both atrophic and neovascular (a more advanced) AMD. The subjects’ mean age was 75.4 years (SD: 2.76, range: 60–97) and 44.6% were female. The criteria for inclusion of AMD patients were: age > 60, AMD diagnosis according to AREDS (Age-Related Eye Disease Study)^54^, and choroidal neovascularization (CNV) and/or geographic atrophy. Diagnosis was also determined according to fluorescein angiogram and optical coherence tomography. Participants were included in all stages of AMD. We excluded individuals with other retinal diseases and individuals with other potential CNV causes such as myopia, trauma, or uveitis. Controls were over the age of 60 with a normal fundus examination and similar systemic exclusion criteria. The study was approved by the institutional ethics committee of Hadassah-Hebrew University Medical Center. All subjects signed informed consent forms that adhered to the tenets of the declaration of Helsinki.

We genotyped all subjects on the custom chip that was developed for the IAMDGC. Genotyping on this chip was performed either via the IAMDGC (at the Center for Inherited Disease Research (Johns Hopkins, USA)) or at the genomics core facility of the Technion (Israel), as previously described^2^. The custom chip, which was previously described, contains ≈ 250,000 tagging markers for imputation and ≈ 250,000 custom markers for AMD^2^. Samples genotyped at either center were merged into a single set and underwent joint imputation, quality control, and further downstream analysis.

We imputed the genomes of our subjects with the following reference panels: the 1000 Genomes Project (1 KG, n = 2504)^55^ and the Ashkenazi Genome Consortium (n = 128)^6^. This strategy was shown to have the highest accuracy for imputing Ashkenazi genomes^56^ and was applied here, given that 60% of our subjects have Ashkenazi ancestry. Unfortunately, a reference panel for non-Ashkenazi Jews or for the non-Jewish populations of Israel does not yet exist, and thus, all samples were imputed with both panels simultaneously. We phased our genomes using SHAPEIT^57,58^ and performed imputation using a standard protocol^58,59^. We describe next the post-imputation quality control (QC) pipeline, as we previously developed^53,60^.

The chip was imputed to 37,126,112 variants. We performed QC according to standard protocols to remove low-quality variants and samples^61^. We excluded variants with imputation quality score R^2^ < 0.6, variants with minor allele frequency < 0.01 (removing 403,979 variants), and variants in Hardy–Weinberg disequilibrium (PLINK 1.9^62,63^). The sex of patients was confirmed using the sex-check option in PLINK. We excluded individuals who were related, having PIHAT > 0.3 in PLINK. We performed principal components analysis (PCA) in PLINK and GCTA^64^ to account for population stratification; the first two principal components were used as covariates in the association analysis (Fig. S1). The final variant count after filtering was 5,353,842 variants in 403 AMD patients and 256 controls.

We performed the discovery GWAS on case–control status using logistic regression in PLINK. To account for population stratification, we used the first two principal components as covariates. The other covariates were age at blood draw and sex. We generated Manhattan and Q–Q plots with *qqman*^65^. For genome-wide significance, we used a P-value threshold of 5 × 10^−8^. To detect suggestive associations, we used a threshold of 5 × 10^−5^. We computed the frequency of risk alleles (either in Europeans or in Ashkenazi Jews) using gnomAD (http://gnomad.broadinstitute.org/) and, if exonic, also in the Exome Variant server (http://evs.gs.washington.edu/EVS). Variants that were outside gene boundaries were reported to nearest gene. Variants contained within a gene were reported with that gene.

To determine whether previously discovered associations replicate in our study, we considered variants within the 34 known loci that were identified in the IAMDGC 2016 GWAS^2^ (Table 5 in the IAMDGC GWAS paper). For each locus (LD block) we retained the variant with the lowest P-value. We considered a nominal significance level of P = 0.05 or a Bonferroni corrected level of P = 0.05/34 = 0.0015.

To test for population-specific replication, we separately studied Ashkenazi Jews (AJ; 242 cases, 136 controls) and Arabs (36 cases and 30 controls). All subjects self-reported their ancestry, and the self-reported ancestry was validated against the genetic ancestry, as determined by their clustering in PCA (Fig. S1). We identified Arab subjects based on self-report (36 cases and 30 controls). We considered all variants in linkage disequilibrium (LD; r^2^ > 0.05 in AJ, using hg19 linkage blocks as per the original Fritsche et al.^2^ paper) to belong to the same locus.

We note that 549/649 of our subjects were part of the original IAMDGC GWAS^2^ (out of a total of 33,976 individuals). Therefore, some degree of replication is expected just by virtue of this sample overlap. However, given that the Israeli samples were less than 2% of the total IAMDGC sample, the effect of the overlap is expected to be small. To validate that, we excluded the Israeli samples from the IAMDGC dataset and re-ran the GWAS analysis (remaining n = 33,515; the “base” study). This created a new, “leave-one-out” GWAS, which did not include any Israeli samples. Indeed, all 34 loci that associated with AMD at genome-wide significance in the original GWAS were also associated with AMD at genome-wide significance in the leave-one-out GWAS.

To replicate putative discoveries from the present study, we recruited additional 155 AMD cases and 69 controls (total 224) according to the same criteria as in the original discovery set. We used this case/control sample to validate the suggestively associated variants from the discovery set. Four variants passed the 5 × 10^−5^ genome-wide threshold in the discovery set, after excluding variants in known AMD risk loci. We genotyped these four variants in our entire replication set using the KASP assay (LGC Group, Middlesex, UK) with custom primers. All heterozygotes were confirmed using Sanger sequencing (Macrogen, Seoul, Korea). We tested the association using EPACTS (https://genome.sph.umich.edu/wiki/EPACTS) and R using two tests. For each SNP, an allelic test compared the proportion of minor alleles between cases and controls. A genotypic test compared the proportion of homozygotes to the minor allele out of all homozygotes between cases and controls.

To generate a polygenic risk score for AMD, we first performed quality control according to standard protocols. We removed variants with strand-ambiguous variants from the base study’s summary statistics. Duplicated variants were removed from both studies, separately. Variants with mismatching alleles were also removed. This has left 4,070,992 overlapping variants between the two studies (directly genotyped or imputed). We then extracted effect sizes from the leave-one-out IAMDGC GWAS, where the Israeli samples were excluded. We used these effect sizes to compute the PRS for individuals in the Israeli study (n = 659; the “target” study).

We generated polygenic risk scores using two approaches for variant selection: clumping and thresholding (C + T) and LDpred2. Briefly, in C + T, index variants are sequentially selected based on having the lowest P-value, and nearby variants in LD with the index variants are removed. Index variants with P-value under a threshold are retained^66,67^. We used PLINK to implement clumping and computed LD (r^2^) using the target study. We set the clumping parameters to r^2^ > 0.5 and ± 500 kb and used nine P-value thresholds: 5 × 10^−8^, and 10^−7^, 10^−6^, 10^−5^, 10^−4^, 0.001, 0.01, 0.1, and 1. The minimum P-value cutoff was set to match the IAMDGC genome-wide significance thresholds.

LDpred2 is a Bayesian method for deriving polygenic scores based on summary statistics while explicitly accounting for LD. Briefly, causal effect sizes are assumed to be a mixture of a normal distribution and a point mass at zero. Posterior mean effects are computed using Gibbs sampling based on the LD matrix and an estimate of the heritability^42,43^. We set the SNP heritability (*h*^*2*^) to 0.47 based on a previous IAMDGC estimate^2^ and used five proportions of causal variants (*p*) spaced on a log scale: 10^−5^, 1.8 × 10^−4^, 0.0032, 0.056, and 1. We also included a third parameter of sparsity (true/false). The analysis was restricted to HapMap3 variants, as recommended by LDpred2 developers. A list of 1,054,330 HapMap3 variants were downloaded from the online repository provided^43^ and 715,011 overlapping variants entered the analysis. The LD matrix was computed using the target study. We used the R package bigsnpr to compute the LD matrix and generate the grid of scores^43^. To avoid confounding by ancestry, in both methods we regressed the scores on the first two principal components and used the residuals as the scores in subsequent analyses.

We used PLINK to calculate PRSs for each of the 659 subjects. Overall, we obtained nine C + T PRSs (nine P-value cutoffs) and ten LDpred2 PRSs, of which four were reported as valid by LDpred2 (P = 0.056, sparse = FALSE; P = 0.056, sparse = TRUE; P = 1, sparse = FALSE; P = 1, sparse = TRUE). To evaluate the accuracy of each score, we used logistic regression of the disease status on the PRS and the following covariates: age, sex, PC1, and PC2. We also included a logistic regression model based on covariates only. We measured the accuracy of each model using the area under the curve (AUC) of the receiver operator characteristic curves (ROC curves), computed using fivefold cross validation. We used the R package *pROC* (https://cran.r-project.org/web/packages/pROC/pROC.pdf) to generate and analyze ROC curves, AUCs, and AUC confidence intervals (ci.auc ()), and the R package *caret* (https://cran.r-project.org/web/packages/caret/vignettes/caret.html) for cross-validation. Individuals with missing age data were excluded from the analysis (four cases and five controls).

We visually inspected the discriminatory power of the PRS using plots of the density of the PRS in cases and controls (using kernel density estimation), and the proportion of AMD cases across quintiles (fifths) of the PRS distribution. Both plots were generated with the R package *ggplot2* (https://cran.r-project.org/web/packages/ggplot2/index.html). Finally, we computed Spearman's rank correlation coefficient to examine the association between the PRS and age at blood draw (as a proxy of the age at diagnosis) among AMD cases. Plots were generated with R package *ggpubr* (https://cran.r-project.org/web/packages/ggpubr/index.html).

### Ethical approval

The study was approved by the institutional ethics committee. All subjects signed informed consent forms that adhered to the tenets of the declaration of Helsinki.

### Supplementary Information

Supplementary Information.

## Data Availability

The full IAMDGC dataset values can be accessed at: http://amdgenetics.org/ including the entire IAMDGC and the Jerusalem dataset specifically. In addition, the GWAS summary statistics and code utilized in this manuscript can be found by contacting the corresponding author via reasonable request. The Related Manuscript Variant supplementary file contains all nomenclature for HGVS for all variants.
