# Supplementary material for: Genome wide association study and genomic risk prediction of age related macular degeneration in Israel
Source: Sci Rep. 2024 Jun 6;14:13034. doi: 10.1038/s41598-024-63065-0 (PMC11156861; doi:10.1038/s41598-024-63065-0)
Supplement: Supplementary file 1 — Supplementary Information. [file 41598_2024_63065_MOESM1_ESM.pdf]

Genome wide association study and genomic risk prediction of age related macular degeneration in Israel

Michelle Grunin[1], Daria Triffon[2], Gala Beykin[1], Elior Rahmani[3], Regev Schweiger[4], Liran Tiosano[1], Samer Khateb[1], Shira Hagbi-Levi[1], Batya Rinsky[1], Refael Munitz[1], Thomas W Winkler[7], Iris M Heid[7], Eran Halperin[4,5,6], Shai Carmi[2*], Itay Chowers[1*]

[1] Department of Ophthalmology, Hadassah-Hebrew University Medical Center, Jerusalem, Israel

[2] Braun School of Public Health and Community Medicine, The Hebrew University of Jerusalem, Jerusalem, Israel

[3] Department of Computational Medicine, University of California, Los Angeles, Los Angeles, CA

[4] Molecular Microbiology and Biotechnology, Tel Aviv University, Tel Aviv, Israel

[5] Department of Anesthesiology, University of California, Los Angeles, Los Angeles, CA, USA

[6] Department of Human Genetics, University of California, Los Angeles, Los Angeles, CA, USA

[7] Department of Genetic Epidemiology, University of Regensburg, Regensburg, Germany

*Indicates equal contribution

Supplementary Material

Supplementary Figures Legends

Figure S1. A PCA plot of the entire discovery cohort. Each symbol represents a single individual. Individuals are color coded based on their self-reported ancestry: Ashkenazi (n=378), North African Sephardi, Turkey, and other Sephardi (n=215), Arab (n=66), and Israel general (n=10). The shape of each symbol corresponds to the AMD PRS quintile (see legend).

Figure S2. A Manhattan plot for the GWAS of AMD in the Israeli discovery cohort (n=659). The X axis indicates chromosomal position, and the Y axis indicates significance, as measured by $-\log_{10}P$.

Figure S3. Accuracy of logistic regression models for predicting AMD disease status using clumping+thresholding (C+T) PRSs. The models also included the following covariates: age, sex, PC1, and PC2. Each curve corresponds to one of nine p-value cutoffs (see legend). The AUC values (after cross-validation) are presented in the legend. CI: confidence interval. Prediction accuracy increased as the p-value threshold decreased.

Figure S4. A scatter plot of age at blood draw and PRS among AMD cases (n=399). We used the best-performing LDpred2 PRS. The PRS was adjusted for PC1 and PC2 to account for confounding by ancestry. The presented age is a proxy for the age at diagnosis. The linear regression parameters are indicated (r=-0.18, P=0.0003). The regression line is also shown, along with the 95% confidence interval (gray band).

Supplementary Tables Legends

Supplementary Table 1: Replication cohort results (n=224) for validation of four top associated SNPs from the AMD GWAS in the Israeli population. Genotype and allelic p-values according to Fisher's exact test are given. The bold bars separate the four SNPs tested for validation. The table provides their genotypes in the replication cohort tested and the p-values.

Supplementary Table 2. Association statistics of 27 variants in 11 known AMD risk loci that replicated in the Israeli discovery set after Bonferroni correction (threshold 0.05/34=0.0015). For each variant, we provide the gene, chromosome (Chr), basepair (BP), odds ratio (OR), 95 percent confidence interval (95% CI) and p-value (P). The variants are sorted by their p-value.

Supplementary Table 3. Association statistics of variants in known AMD risk loci that were nominally associated with AMD in the Israeli discovery set (P<0.05, 31/34 loci).

Supplementary Table 4. Association statistics of variants in known AMD risk loci in the Ashkenazi subpopulation (P<1x10^{-4}). The data is reported as in Supplementary Table 3.

Supplementary Table 5 Association statistics of variants in known AMD risk loci in the Arab subpopulation ($P<1x10^{-4}$). The data is reported as in Supplementary Table 3.

Supplementary Table 6: Association statistics of the specific 52 variants present in Fritsche et al, 2016, Nature Genetics as most significant in the European population, with p-values from the Israeli GWAS.

Supplementary Table 7: Eigenvalues from principal components to determine the most informative (PC 1+2)

Supplementary Table 8: Genotypic and allelic p-values for the Discovery Set for the 4 SNPs analyzed in replication. Genotype and allelic p-values according to Fisher's exact test are given. The bold bars separate the four SNPs tested for validation. The table provides their genotypes in the discovery cohort tested and the p-values.

Figure S1:

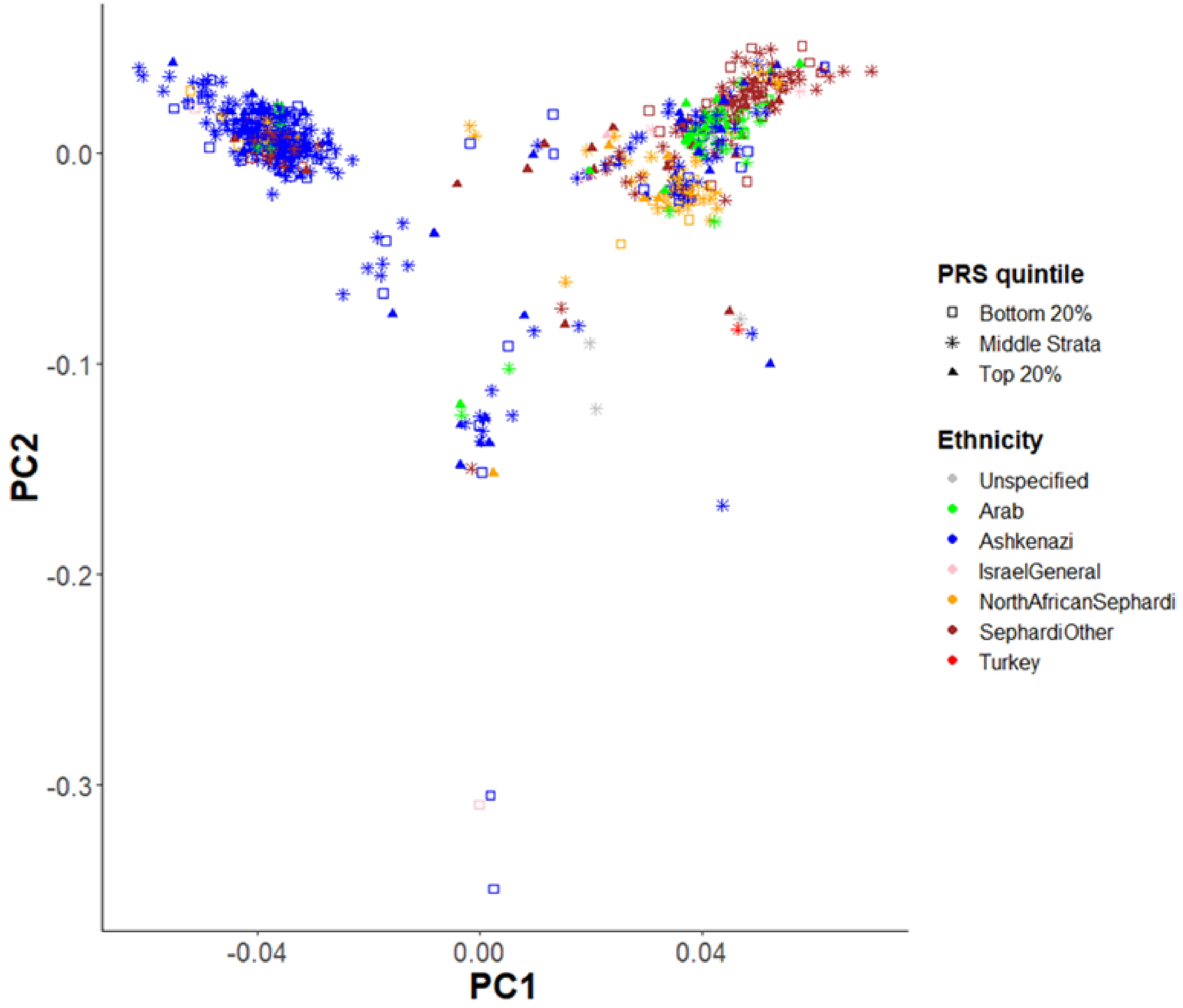

Figure S2:

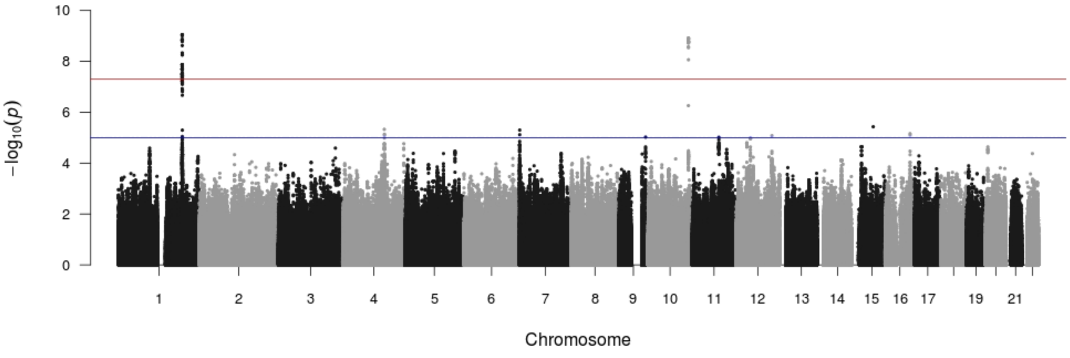

Figure S3:

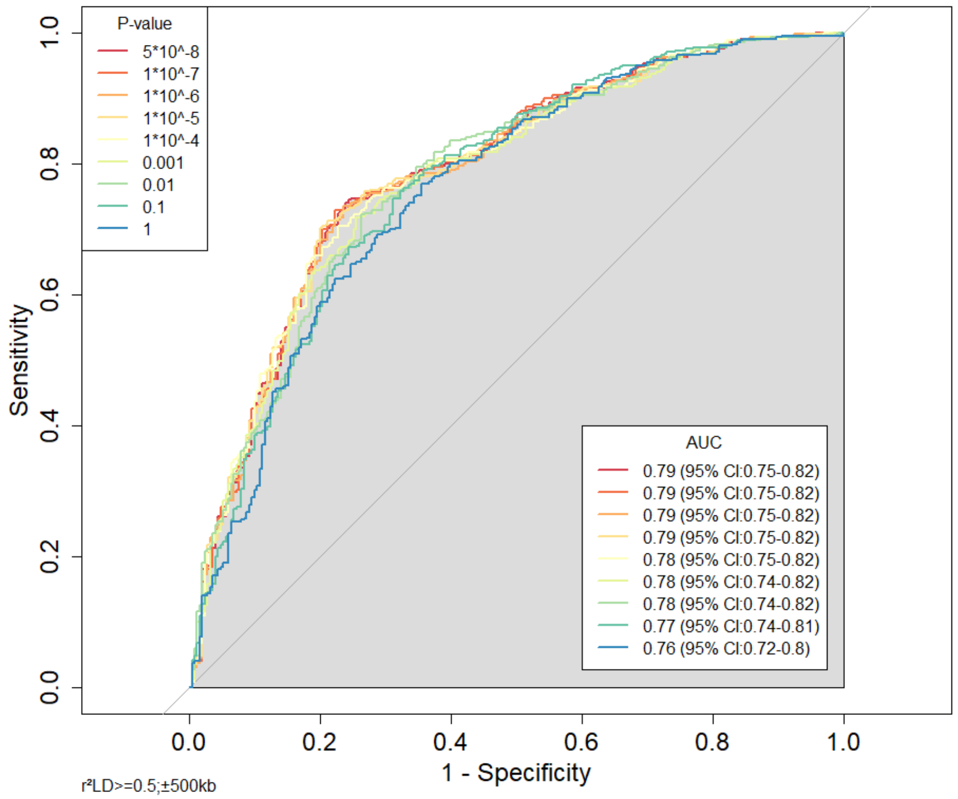
P-value
5*10^-8
1*10^-7
1*10^-6
1*10^-5
1*10^-4
0.001
0.01
0.1
1
AUC
0.79 (95% CI:0.75-0.82)
0.79 (95% CI:0.75-0.82)
0.79 (95% CI:0.75-0.82)
0.79 (95% CI:0.75-0.82)
0.78 (95% CI:0.75-0.82)
0.78 (95% CI:0.74-0.82)
0.78 (95% CI:0.74-0.82)
0.77 (95% CI:0.74-0.81)
0.76 (95% CI:0.72-0.8)
Sensitivity
1 - Specificity
0.0
0.2
0.4
0.6
0.8
1.0
r²LD>=0.5;±500kb

Figure S4:

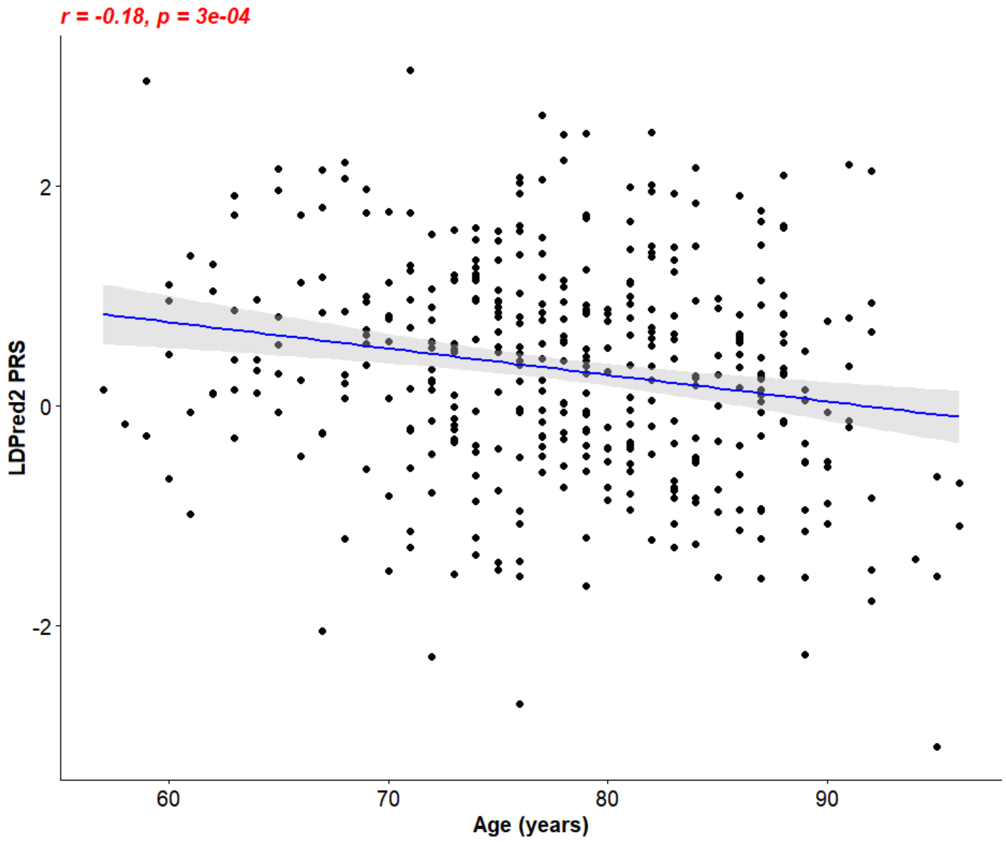

Supplementary Tables

Supplementary Table 1:

**rs12701455**<br><br>**Chromosome** 7- **BP**<br><br>1055409	**Homozygote**<br><br>**Major**	**Heterozygote**	**Homozygote**<br><br>**Minor**	**p-value**<br><br>**(Genotype)**	**p-value**<br><br>**(Allele)**
**AMD**	40	1	3	0.0287	0.53
**Control**	35	6	0		

**rs116928937** Chromosome 15 – **BP** 65677446	**Homozygote Major**	**Heterozygote**	**Homozygote Minor**	**p-value (Genotype)**	**p-value (Allele)**
**AMD**	108	4	0	0.92	1
**Control**	73	2	0		

**rs1506825** Chromosome 16 – **BP** 76483019	**Homozygote Major**	**Heterozygote**	**Homozygote Minor**	**p-value (Genotype)**	**p-value (Allele)**
**AMD**	59	66	38	0.85	0.34
**Control**	25	29	20		

**rs1195500** Chromosome 15 – **BP** 29687047	**Homozygote Major**	**Heterozygote**	**Homozygote Minor**	**p-value (Genotype)**	**p-value (Allele)**
**AMD**	110	37	8	<0.0001	<0.0001
**Control**	31	24	14		

Supplementary Table 2:

	**Gene**	**Chr**	**BP**	**OR**	**95%CI**	**P**

1	CFH	1	196695161	0.4687	[0.25-0.85]	1.56E-09
2	ARMS2	10	124214448	2.071	[1.16-3.84]	3.424E-09
3	HTRA1	10	124221270	2.061	[1.15-3.79]	5.077E-09
4	CFHR5	1	196978615	0.5161	[0.29-0.89]	5.195E-08
5	CFHR2	1	196927791	1.894	[1.11-3.39]	1.163E-07
6	CFHR4	1	196870299	0.436	[0.21-0.96]	0.00000762
7	KCNT2	1	196406715	0.5845	[0.33-0.96]	0.00001443
8	F13B	1	197012111	1.7	[1.03-2.83]	0.00001487
9	SYN3	22	33047598	0.3696	[0.13-0.97]	0.00005685
10	ASPM	1	197094030	1.616	[1.01-2.62]	0.0000688

11	ZBTB41	1	197132378	0.4372	[0.19-0.99]	0.0000732
12	PLEKHA1	10	124148167	0.6403	[0.39-0.98]	0.0001034
13	VPS29	12	110935268	0.5591	[0.31-1.01]	0.000218
14	CRB1	1	197199434	1.568	[0.99-2.5]	0.0002242
15	PPTC7	12	110994068	0.5699	[0.24-1.01]	0.0002391
16	ZNF557	19	7083629	1.576	[0.98-2.51]	0.0002624
17	HVCN1	12	111099721	0.5184	[0.26-1.03]	0.0004554
18	MICALL1	22	38318897	1.48	[0.97-2.25]	0.0007491
19	SMC5	9	72920724	0.427	[0.17-1.06]	0.0008093
20	PPP1CC	12	111160003	0.5937	[0.34-1.04]	0.0008394

21	TCTN1	12	111081197	0.5847	[0.32-1.04]	0.0008647
22	HLA-B	6	31324864	0.4779	[0.22-1.06]	0.0008669
23	RAD9B	12	110948906	0.5532	[0.29-1.0504]	0.001058
24	EIF3L	22	38273303	1.476	[0.97-2.25]	0.001161
25	TTC23L	5	34840841	0.6909	[0.46-1.033]	0.001183
26	ACHE	7	100491753	0.1589	[0.02-1.18]	0.001267
27	KIAA0100	17	26956537	0.6755	[0.44-1.04]	0.001267

Supplementary Table 3:

AMD Locus According to Fritsche et al 2016	Approved Symbol (HGNC)	P-value
1	*CFH*	1.56E-09
18	*ARMS2*	3.424E-09
18	*HTRA1*	5.077E-09
1	*CFHR5*	5.195E-08

1	*CFHR2*	1.163E-07
1	*CFHR4*	0.00000762
1	*KCNT2*	0.00001443
1	*F13B*	0.00001487
33	*SYN3*	0.00005685
1	*ASPM*	0.0000688
1	*ZBTB41*	0.0000732
18	*PLEKHA1*	0.0001034
1	*CRB1*	0.0001282
20	*PPTC7*	0.0001725
28	*ZNF557*	0.0001836
20	*VPS29*	0.000218
20	*HVCN1*	0.0004554
34	*MICALL1*	0.0007491
14	*SMC5*	0.0008093
20	*PPP1CC*	0.0008394
20	*TCTN1*	0.0008647
8	*HLA-B*	0.0008669
20	*RAD9B*	0.001058
34	*EIF3L*	0.001161
7	*TTC23L*	0.001183
11	*ACHE*	0.001267
26	*KIAA0100*	0.001267
3	*ADAMTS9-AS2*	0.001549
20	*FAM216A*	0.001563
8	*VARS*	0.001596
8	*C6orf48*	0.00162
10	*LHFPL3*	0.001763
18	*TACC2*	0.002007
34	*GCAT*	0.002065
7	*RAI14*	0.002168
8	*PRRC2A*	0.002247
8	*LSM2*	0.002253
27	*ACTG1*	0.002291
8	*SLC44A4*	0.002362
30	*TOMM40*	0.002687
5	*EGF*	0.002703
22	*ZFP36L1*	0.002801
8	*MSH5*	0.002847

8	*MSH5-SAPCD1*	0.002847
7	*LMBRD2*	0.002997
17	*KIAA1217*	0.00307
8	*BAG6*	0.003264
8	*ABHD16A*	0.003366
10	*SRPK2*	0.003392
8	*C6orf47*	0.003486
8	*GPANK1*	0.003486
8	*LY6G5C*	0.003486
8	*CSNK2B*	0.003674
31	*SLC12A5*	0.003686
23	*SLTM*	0.00382
8	*SAPCD1*	0.003898
33	*TIMP3*	0.003935
5	*COL25A1*	0.004167
34	*BAIAP2L2*	0.004208
19	*DGKA*	0.004239
8	*EHMT2*	0.004284
8	*VWA7*	0.004415
28	*ZNRF4*	0.004423
20	*DTX1*	0.004452
8	*C2*	0.004495
28	*RFX2*	0.004542
12	*TNFRSF10B*	0.004838
28	*INSR*	0.004913
5	*ELOVL6*	0.004992
30	*APOC1*	0.005035
19	*WIBG*	0.005741
8	*LY6G6F*	0.005813
25	*GABARAPL2*	0.006067
8	*HCG26*	0.006204
30	*PVRL2*	0.006553
2	*RHBDD1*	0.006662
28	*ACER1*	0.006821
25	*ADAT1*	0.006832
10	*ATXN7L1*	0.007322
8	*NCR3*	0.00746
31	*CDH22*	0.007473
26	*PIGS*	0.007713

28	*DUS3L*	0.007766
34	*H1F0*	0.007772
34	*C22orf23*	0.007828
8	*ATP6V1G2*	0.008046
8	*ATP6V1G2-DDX39B*	0.008046
8	*NFKBIL1*	0.008046
21	*TEX26*	0.008121
11	*GIGYF1*	0.008345
25	*CHST5*	0.008811
4	*COL8A1*	0.008929
4	*MIR548G*	0.008929
25	*KARS*	0.008961
30	*GEMIN7*	0.009067
23	*AQP9*	0.009112
24	*DOK4*	0.009633
8	*MICA*	0.009687
29	*SBNO2*	0.009692
34	*SOX10*	0.009929
12	*RHOBTB2*	0.009956
29	*HMHA1*	0.009991
26	*NEK8*	0.009999
9	*CAPN11*	0.01005
7	*DNAJC21*	0.01008
26	*SDF2*	0.01016
28	*PRR22*	0.0104
34	*POLR2F*	0.0105
30	*ZNF285*	0.01068
2	*COL4A4*	0.01086
3	*PRICKLE2*	0.01118
28	*CATSPERD*	0.01123
19	*CD63*	0.01133
8	*TCF19*	0.01249
8	*HLA-DQB2*	0.01254
8	*PSORS1C1*	0.01291
23	*FAM63B*	0.01298
2	*COL4A3*	0.01309
14	*TRPM3*	0.0132
8	*AIF1*	0.01348
3	*ADAMTS9*	0.0135

24	*CPNE2*	0.01373
7	*SKP2*	0.01374
25	*CTRB1*	0.01374
25	*TMEM231*	0.01388
22	*RAD51B*	0.01394
8	*SKIV2L*	0.01439
32	*PCK1*	0.01459
26	*SUPT6H*	0.01473
30	*KLC3*	0.01474
4	*CMSS1*	0.01478
4	*FILIP1L*	0.01478
20	*OAS1*	0.01493
8	*BTNL2*	0.01543
20	*CCDC63*	0.01593
2	*AGFG1*	0.01644
28	*SAFB*	0.01649
8	*C6orf10*	0.01673
11	*AZGP1*	0.01691
8	*HCG23*	0.01694
32	*APCDD1L*	0.01701
24	*SLC12A3*	0.01709
8	*ABCF1*	0.01727
19	*PAN2*	0.01752
10	*PUS7*	0.01772
5	*CCDC109B*	0.01785
29	*TMEM259*	0.01813
11	*EPO*	0.01823
23	*LIPC*	0.01831
30	*CLASRP*	0.01835
6	*DAB2*	0.01893
21	*B3GALTL*	0.01913
21	*TEX26-AS1*	0.01938
19	*CS*	0.01939
8	*TAP2*	0.01974
30	*PVR*	0.01982
17	*GPR158*	0.01988
3	*MIR548A2*	0.01991
23	*ADAM10*	0.01999
11	*ZNF3*	0.02024

18	*DMBT1*	0.02062
8	*CCHCR1*	0.02071
25	*BCAR1*	0.02125
25	*TERF2IP*	0.02145
8	*MRPS18B*	0.02146
29	*GRIN3B*	0.02167
30	*ERCC2*	0.02194
10	*KMT2E-AS1*	0.0221
27	*ASPSCR1*	0.02225
29	*C19orf26*	0.02243
25	*CTRB2*	0.02263
20	*CUX2*	0.02269
8	*CDSN*	0.02282
29	*ABCA7*	0.02311
10	*KMT2E*	0.02314
22	*ACTN1*	0.02375
28	*C3*	0.02377
26	*NLK*	0.02403
19	*RNF41*	0.02412
8	*MICB*	0.02445
28	*LONP1*	0.02503
29	*PRSS57*	0.02507
10	*LINC01004*	0.02536
27	*HGS*	0.02542
33	*RTCB*	0.02543
11	*AZGP1P1*	0.02547
31	*PCIF1*	0.02556
7	*PRLR*	0.02592
8	*NOTCH4*	0.02598
30	*CBLC*	0.02617
25	*CHST6*	0.0264
6	*FYB*	0.02651
29	*CNN2*	0.02674
27	*SLC25A10*	0.02679
24	*CX3CL1*	0.02692
7	*CAPSL*	0.02764
8	*PPT2-EGFL8*	0.02765
8	*NRM*	0.02772
34	*KCNJ4*	0.02772

7	*IL7R*	0.02779
30	*IGSF23*	0.02782
8	*FLOT1*	0.02893
29	*AZU1*	0.02942
19	*ANKRD52*	0.02947
11	*PILRA*	0.02955
8	*ATAT1*	0.02962
11	*EPHB4*	0.02972
31	*PLTP*	0.02988
27	*CCDC137*	0.03008
27	*OXLD1*	0.03008
29	*STK11*	0.03014
20	*BRAP*	0.03019
19	*RDH5*	0.03027
12	*LOXL2*	0.03037
28	*PTPRS*	0.03047
7	*NADK2*	0.03069
17	*PRTFDC1*	0.03085
19	*RAB5B*	0.03124
26	*PROCA1*	0.0315
26	*NOS2*	0.03158
27	*NPLOC4*	0.03158
33	*SLC5A4*	0.03166
12	*CHMP7*	0.03172
7	*SPEF2*	0.03181
20	*ACAD10*	0.03248
11	*ZAN*	0.03257
14	*KLF9*	0.03261
8	*MDC1*	0.03274
26	*KRT18P55*	0.03309
8	*PPP1R18*	0.03342
24	*MT1H*	0.03343
28	*KHSRP*	0.03348
26	*POLDIP2*	0.03395
18	*BTBD16*	0.03403
34	*CARD10*	0.03447
11	*MCM7*	0.03451
20	*PTPN11*	0.03456
30	*NKPD1*	0.03472

24	*CCL22*	0.03481
6	*C9*	0.03496
30	*RSPH6A*	0.0353
11	*TFR2*	0.03558
34	*CDC42EP1*	0.03579
27	*PCYT2*	0.0359
1	*DENND1B*	0.03598
9	*TMEM63B*	0.03603
26	*SARM1*	0.03622
7	*UGT3A2*	0.03637
34	*NOL12*	0.03656
24	*POLR2C*	0.03659
28	*EMR1*	0.03659
24	*PLLP*	0.03674
24	*MT1G*	0.03684
8	*HLA-C*	0.03748
8	*ATF6B*	0.03778
12	*PEBP4*	0.03791
31	*ZNF335*	0.03801
8	*GNL1*	0.03826
7	*RANBP3L*	0.03875
24	*RSPRY1*	0.03892
33	*BPIFC*	0.03897
33	*RFPL3*	0.03904
19	*SUOX*	0.03917
32	*RAB22A*	0.0393
30	*CKM*	0.03938
29	*HCN2*	0.03973
30	*PPP1R37*	0.03975
34	*ELFN2*	0.03983
26	*FAM222B*	0.04021
8	*TAP1*	0.04025
19	*SARNP*	0.04059
24	*OGFOD1*	0.04059
34	*SLC16A8*	0.04085
19	*OR6C4*	0.04093
29	*MIDN*	0.04104
25	*CFDP1*	0.04105
18	*NSMCE4A*	0.04152

22	*DCAF5*	0.04154
20	*NAA25*	0.04157
19	*SLC39A5*	0.0417
31	*NCOA5*	0.04177
30	*DMWD*	0.04185
24	*FAM192A*	0.04223
28	*CLPP*	0.04227
20	*OAS2*	0.04236
2	*C2orf83*	0.0427
11	*GAL3ST4*	0.04306
7	*UGT3A1*	0.04318
8	*LTA*	0.04333
8	*HCG27*	0.04337
26	*FLOT2*	0.04352
23	*ALDH1A2*	0.0439
25	*TMEM170A*	0.0439
24	*NLRC5*	0.04394
23	*HSP90AB4P*	0.04408
12	*TNFRSF10C*	0.04477
26	*SPAG5*	0.04481
5	*PLA2G12A*	0.04484
28	*FUT3*	0.04519
20	*SH2B3*	0.04551
17	*ARHGAP21*	0.04561
4	*DCBLD2*	0.0459
8	*MUC22*	0.04609
25	*ZFP1*	0.04623
32	*PMEPA1*	0.04633
18	*FAM24B*	0.04651
8	*HCP5*	0.04676
5	*CFI*	0.04735
34	*TRIOBP*	0.04743
5	*CASP6*	0.04754
29	*WDR18*	0.04767
8	*PPP1R10*	0.04797
20	*ATXN2*	0.04812
26	*FOXN1*	0.04815
10	*EFCAB10*	0.04819
11	*SLC12A9*	0.04846

27	*BAHCC1*	0.04868
25	*WDR59*	0.04878
6	*OSMR*	0.0488
25	*MLKL*	0.04883
32	*VAPB*	0.04886
8	*HLA-DPA1*	0.04963
8	*HLA-DPB1*	0.04963
8	*PRR3*	0.04968

Supplementary Table 4:

	**Gene**	**Chr**	**BP**	**OR**	**95%CI**	**P**
1	ZNF180	19	44983567	0.2176	[0.04-1.14]	0.001235
2	CFHR5	1	196962502	0.2461	[0.05-1.16]	0.001626
3	GPR128	3	100396915	0.1992	[0.03-1.2]	0.002002
4	KCNT2	1	196348779	0.2226	[0.04-1.2]	0.002353
5	SYN3	22	33001207	5.271	[0.04-1.35]	0.002511
6	LHFPL3	7	104245132	0.1481	[0.01-1.31]	0.003227
7	PSORS1C3	6	31141523	0.2548	[0.05-1.22]	0.003532
8	CDC42EP1	22	37958163	3.541	[0.8-11.68]	0.003643
9	RAB5B	12	56389293	0.3115	[0.08-1.2]	0.003893
10	CMSS1	3	99683653	0.1851	[0.02-1.3]	0.004253
11	FILIP1L	3	99683653	0.1851	[0.02-1.3]	0.004253

12	MIR548G	3	99683653	0.1851	[0.02-1.3]	0.004253
13	RPS26	12	56435929	0.323	[0.02-1.85]	0.004307
14	SUOX	12	56393337	0.3192	[0.52-1.3]	0.004432

Supplementary Table 5:

	Gene	Chr	BP	OR	95%CI	P
1	HTRA1	10	124221270	2.221	[1.08-4.58]	2.914E-06
2	ARMS2	10	124215211	2.23	[1.08-4.6]	3.062E-06
3	CFH	1	196673430	1.984	[1.02-3.84]	0.00003481
4	CFHR2	1	196927791	1.931	[1.01-3.68]	0.00006749
5	ZNF557	19	7083629	1.823	[0.98-3.4]	0.0003984
6	SRPK2	7	104877373	0.5753	[0.32-1.03]	0.0005616
7	TOMM40	19	45396219	-3.424	[0.25-1.04]	0.0006182
8	CFHR5	1	196978615	0.582	[0.32-1.03]	0.0007713
9	HLA-B	6	31324864	0.3546	[0.11-1.08]	0.0009341
10	CFHR4	1	196870299	0.3862	[0.14-1.07]	0.0009646
11	PLEKHA1	10	124139393	1.695	[0.95-3]	0.0009873

Related Manuscript Variant File: Contains nomenclature for every variant listed in this paper according to HGVS, checked with Mutalyzer.

dbSNP Identifier	HGVS nomenclature	Chr:BP
rs1195500:G>A,C,T	NC_000015.10:g.29394842G>A, NC_000015.10:g.29394842G>C, NC_000015.10:g.29394842G>T	Chr15:29687047 (GRCh37)
rs116928937:C>A	NC_000015.10:g.65385107C>A	Chr15:65677446 (GRCh37)
rs142491581:C>G	NC_000004.12:g.127714391C>G	Chr4:128635546 (GRChr37)
rs6449549:C>T	NC_000005.10:g.61734604C>T	Chr5:61030432 (GRChr37)
rs4235321:G>A	NC_000004.12:g.24954305G>A	Chr4:24955928 (GRChr37)
rs41592:G>A	NC_000007.14:g.83012543G>A	Chr7:82641860 (GRChr37)
rs12701455:A>G,T	NC_000007.14:g.1015773A>G,NC_000007.14:g.1015773A>T	Chr7:1055409 (GRChr37)
rs11689931:T>G	NC_000002.12:g.205576255T>G	Chr2:206440979 (GRChr37)
rs1506825:A>C,T	NC_000016.10:g.76449122A>C,NC_000016.10:g.76449122A>T	Chr16:76483019 (GRChr37)

Gene	dbSNP TopVariant	HGVS	TopVariant:Chr:BP (GRChr37)
CFH	rs3766405:C>A,T	NC_000001.11:g.196726031C>A,NC_000001.11:g.196726031C>T	Chr1:196695161
ARMS2	rs10490924:G>C,T	NC_000010.11:g.122454932G>C,NC_000010.11:g.122454932G>T	Chr10:124214448
HTRA1	rs1049331:C>T	NC_000010.11:g.122461753C>T	Chr10:124221270
CFHR5	rs10922153:T>G	NC_000001.11:g.197009484T>G	Chr1:196978615
CFHR2	rs2026547:G>A	NC_000001.11:g.196958661G>A	Chr1:196927791

CFHR4	rs34833349:A>G	NC_000001.11:g.196901169A>G	Chr1:196870299
KCNT2	rs10922068:T>A,C	NC_000001.11:g.196437585T>A,NC_000001.11:g.196437585T>C	Chr1:196406715
F13B	rs10754210:G>A	NC_000001.11:g.197042981G>A	Chr1:197012111
SYN3	rs5754187:C>T	NC_000022.11:g.32651611C>T	Chr22:33047598
ASPM	rs6676084:C>T	NC_000001.11:g.197124900C>T	Chr1:197094030
ZBTB41	rs4350226:G>A,C,T	NC_000001.11:g.197163247G>A,NC_000001.11:g.197163247G>C,NC_000001.11:g.197163247G>T	Chr1:197132378
PLEKHA1	rs2421017:A>G	NC_000010.11:g.122388651A>G	Chr10:124148167
VPS29	rs184629901:T>C	NC_000012.12:g.110497463T>C	Chr12:110935268
CRB1	rs12737179:T>C,G	NC_000001.11:g.197230303T>C,NC_000001.11:g.197230303T>G	Chr1:197199434
PPTC7	rs56159960:T>C	NC_000012.12:g.110556262T>C	Chr12:110994068
ZNF557	rs966591:A>C,G	NC_000019.10:g.7083618A>C, NC_000019.10:g.7083618A>G	Chr19: 7083629
HVCN1	rs73191857:C>T	NC_000012.12:g.110661916C>T	Chr12: 111099721
MICALL1	rs9607501:G>A,C,T	NC_000022.11:g.37922890G>A,NC_000022.11:g.37922890G>C,NC_000022.11:37922890G>T	Chr22: 38318897
SMC5	rs66524845:A>G	NC_000009.12:g.70305808A>G	Chr9: 72920724
PPP1CC	rs1973505:G>A	NC_000012.12:g.110722198G>A	Chr12: 111160003
TCTN1	rs7953794:A>G	NC_000012.12:g.110643392A>G	Chr12: 111081197
HLA-B	rs151341076:G>A,C,T	NC_000006.12:g.31357087G:A, NC_000006.12:g.31357087G>	Chr6:31324864

		C,NC_000006.12:g.31357087G>T	
CFHR5	rs7547265:G>C,T	NC_000001.11:g.196993372G>C,NC_000001.11:g.196993372G>T	Chr1: 196962502
GPR128	rs7629279:G>T	NC_000003.12:g.100678070G>T	Chr3:100396915
KCNT2	rs7527415:C>T	NC_000001.11:g.196379649C>T	Chr1:196348779
LHFPL3	rs17139096:A>G,T	NC_000007.14:g.104604684A>G,NC_000007.14:g.104604684A>T	Chr7:104245132
PSORS1C3	rs887468:C>T	NC_000006.12:g.31173746C>T	Chr6:31141523
CDC42EP1	rs2235335:G>A,C	NC_000022.11:g.37562156G>A,NC_000022.11:g.37562156G>C	Chr22:37958163
RAB5B	rs705700:T>A,C	NC_000012.12:g.55995509T>A,NC_000012.12:g.55995509T>C	Chr12:56389293
FILIP1L	rs73138610:C>A,G	NC_000003.12:g.99964809C>A,NC_000003.12:g.99964809C>G	Chr3:99683653
RPS26	rs1131017:C>A,G,T	NC_000012.12:g.56042145C>A,NC_000012.12:g.56042145C>G,NC_000012.12:g.56042145C>T	Chr12:56435929
SUOX	rs1081975:C>A,G	NC_000012.12:g.55999553C>A,NC_000012.12:g.55999553C>G	Chr12:56393337
HTRA1	rs1049331:C>T	NC_000010.11:g.122461753C>T	Chr10:124221270
ARMS2	rs36212733:T>A,C,G	NC_000010.11:g.122455695T>A,NC_000010.11:g.122455695T>C, NC_000010.11:g.122455695T>G	Chr10:124215211
CFH	rs9970075:T>A,G	NC_000001.11:g.196704299T>A,NC_000001.11:g.196704299T>G	Chr1:196673430

CFHR2	rs2026547:G>A	NC_000001.11:g.196958661G>A	Chr1:196927791
ZNF557	rs966591:A>C,G	NC_000019.10:g.7083618A>C, NC_000019.10:g.7083618A>G	Chr19:7083629
SRPK2	rs6950104:G>A,C	NC_000007.14:g.105236926G>A,NC_000007.14:g.105236926G>C	Chr7:104877373
TOMM40	rs157582:C>T	NC_000019.10:g.44892961C>T	Chr19:45396219
CFHR5	rs10922153:T>G	NC_000001.11:g.197009484T>G	Chr1:196978615
HLA-B	rs151341076:G>A,C,T	NC_000006.12:g.31357087G>A,NC_000006.12:g.31357087G>C, NC_000006.12:g.31357087G>T	Chr6:31324864
CFHR4	rs34833349:A>G	NC_000001.11:g.196901169A>G	Chr1:196870299
PLEKHA1	rs11200594:C>G,T	NC_000010.11:g.122379876C>G,NC_000010.11:g.122379876C>T	Chr10:124139393

Supplementary Table 6:

**Lead Variant**	**Chr**	**Positiona**	**Major/minor allele**	**Locus name**	**P-value Israel**
rs10922109	1	196704632	C/A	*CFH*	9.274E-10
rs62247658	3	64715155	T/C	*ADAMTS9-AS2*	0.7706
rs140647181	3	99180668	T/C	*COL8A1*	NA
rs10033900	4	110659067	C/T	*CFI*	0.5706
rs62358361	5	39327888	G/T	*C9*	NA
rs116503776	6	31930462	G/A	*C2/CFB/SKIV2L*	0.09955
rs943080	6	43826627	T/C	*VEGFA*	0.8183
rs79037040	8	23082971	T/G	*TNFRSF10A*	NA
rs1626340	9	101923372	G/A	*TGFBR1*	NA

rs3750846	10	124215565	T/C	*ARMS2/HTRA1*	1.71E-09
rs9564692	13	31821240	C/T	*B3GALTL*	
rs61985136	14	68769199	T/C	*RAD51B*	0.8228
rs2043085	15	58680954	T/C	*LIPC*	0.06453
rs5817082	16	56997349	C/CA	*CETP*	NA
rs2230199	19	6718387	C/G	*C3*	0.2837
rs429358	19	45411941	T/C	*APOE*	0.03936
rs5754227	22	33105817	T/C	*SYN3/TIMP3*	0.655
rs8135665	22	38476276	C/T	*SLC16A8*	0.143
rs11884770	2	228086920	C/T	*COL4A3*	0.9023
rs114092250	5	35494448	G/A	*PRLR/SPEF2*	NA
rs7803454	7	99991548	C/T	*PILRB/PILRA*	0.253
rs1142	7	104756326	C/T	*KMT2E/SRPK2*	0.1553
rs71507014	9	73438605	GC/G	*TRPM3*	
rs10781182	9	76617720	G/T	*MIR6130/RORB*	0.0736
rs2740488	9	107661742	A/C	*ABCA1*	NA
rs12357257	10	24999593	G/A	*ARHGAP21*	0.9744
rs3138141	12	56115778	C/A	*RDH5/CD63*	NA
rs61941274	12	112132610	G/A	*ACAD10*	NA
rs72802342	16	75234872	C/A	*CTRB2/CTRB1*	0.3293
rs11080055	17	26649724	C/A	*TMEM97/VTN*	0.3476
rs6565597	17	79526821	C/T	*NPLOC4/TSPAN10*	NA
rs67538026	19	1031438	C/T	*CNN2*	NA
rs142450006	20	44614991	TTTTC/T	*MMP9*	NA
rs201459901	20	56653724	T/TA	*C20orf85*	NA

Supplementary Table 7:

Eigenvalues
4.70301
2.44298
2.21493
1.92812
1.77873
1.67937
1.66089
1.61673
1.603
1.56959
1.55571
1.52121

1.51469
1.46911
1.41975
1.40046
1.38949
1.37321
1.3683
1.35253

Supplementary Table 8:

**rs12701455** **Chromosome** 7- **BP** 1055409	**Homozygote Major**	**Heterozygote**	**Homozygote Minor**	**p-value (Genotype)**	**p-value (Allele)**
**AMD**	280	102	6	0.0014	<0.0001
**Control**	137	99	13		
**rs116928937** **Chromosome** 15 – **BP** 65677446	**Homozygote Major**	**Heterozygote**	**Homozygote Minor**	**p-value (Genotype)**	**p-value (Allele)**
**AMD**	396	7	0	<0.0001	<0.0001
**Control**	228	28	0		
**rs1506825** **Chromosome** 16 – **BP** 76483019	**Homozygote Major**	**Heterozygote**	**Homozygote Minor**	**p-value (Genotype)**	**p-value (Allele)**
**AMD**	124	201	74	<0.0001	<0.0001
**Control**	43	134	79		
**rs1195500**	**Homozygote Major**	**Heterozygote**	**Homozygote Minor**	**p-value (Genotype)**	**p-value**

**Chromosome** 15 – **BP** 29687047					**(Allele)**
**AMD**	297	94	4	0.001	<0.0001
**Control**	148	89	12		
